# Association of high pressure and alkaline condition for solubilization of inclusion bodies and refolding of the NS1 protein from zika virus

**DOI:** 10.1186/s12896-018-0486-2

**Published:** 2018-12-12

**Authors:** Cleide Mara Rosa da Silva, Rosa Maria Chura-Chambi, Lennon Ramos Pereira, Yraima Cordeiro, Luís Carlos de Souza Ferreira, Ligia Morganti

**Affiliations:** 1 Instituto de Pesquisas Energéticas e Nucleares, IPEN-CNEN/SP, Centro de Biotecnologia, Av. Prof. Lineu Prestes, 2242, São Paulo, 05508-000 Brazil; 2 Departamento de Microbiologia, Universidade de São Paulo, Instituto de Ciências Biomédicas, Av. Prof. Lineu Prestes ,1374, São Paulo, 05508-000 Brazil; 3 Universidade Federal do Rio de Janeiro, Faculdade de Farmácia, Av. Carlos Chagas Filho 373, Rio de Janeiro, 21941-902 Brazil

**Keywords:** Protein refolding, Inclusion bodies, NS1, Dengue virus and Zika virus, High hydrostatic pressure, Alkaline pH

## Abstract

**Background:**

Proteins in inclusion bodies (IBs) present native-like secondary structures. However, chaotropic agents at denaturing concentrations, which are widely used for IB solubilization and subsequent refolding, unfold these secondary structures. Removal of the chaotropes frequently causes reaggregation and poor recovery of bioactive proteins. High hydrostatic pressure (HHP) and alkaline pH are two conditions that, in the presence of low level of chaotropes, have been described as non-denaturing solubilization agents. In the present study we evaluated the strategy of combination of HHP and alkaline pH on the solubilization of IB using as a model an antigenic form of the zika virus (ZIKV) non-structural 1 (NS1) protein.

**Results:**

Pressure-treatment (2.4 kbar) of NS1-IBs at a pH of 11.0 induced a low degree of NS1 unfolding and led to solubilization of the IBs, mainly into monomers. After dialysis at pH 8.5, NS1 was refolded and formed soluble oligomers. High (up to 68 mg/liter) NS1 concentrations were obtained by solubilization of NS1-IBs at pH 11 in the presence of arginine (Arg) with a final yield of approximately 80% of total protein content. The process proved to be efficient, quick and did not require further purification steps. Refolded NS1 preserved biological features regarding reactivity with antigen-specific antibodies, including sera of ZIKV-infected patients. The method resulted in an increase of approximately 30-fold over conventional IB solubilization-refolding methods.

**Conclusions:**

The present results represent an innovative non-denaturing protein refolding process by means of the concomitant use of HHP and alkaline pH. Application of the reported method allowed the recovery of ZIKV NS1 at a condition that maintained the antigenic properties of the protein.

**Electronic supplementary material:**

The online version of this article (10.1186/s12896-018-0486-2) contains supplementary material, which is available to authorized users.

## Background

Inclusion bodies (IBs) are composed of both active and inactive polypeptides. Polypeptides in IBs exhibit both properly folded domains, which account for the native-like structure and misfolded stretches, which are responsible for non-native intermolecular β-sheet organization that supports the IB architecture [1, 2]. The IBs produced by transformed bacteria can thus present a certain percentage of proteins with native-like structure and even biological activity [3]. The classical recovery of biologically active proteins from IBs involves refolding by solubilization of protein aggregates under denaturing conditions. However, significant reaggregation often occurs once the concentration of the denaturant agent goes below a critical level [4]. A method for reducing reaggregation is the use of a solubilization process that avoids complete denaturation and exposure of the hydrophobic patches, which can be achieved using mild conditions without generation of unfolded domains As an example of these mild conditions is the utilization of HHP at up to 3 kbar that solubilizes IBs by diminishing hydrophobic and electrostatic interactions and does not induce loss of secondary structure [5, 6]. There are some reports about the use of HHP (1–3 kbar) for dissociation of IBs and protein refolding. However, chaotropic reagents are frequently added to improve the efficacy of solubilization, although at lower concentrations than those used in conventional refolding protocols [7–11].

Alkaline pH can solubilize aggregated proteins by electrostatic repulsion, a condition that is less denaturing than high levels of chaotropic reagents [12]. High pH has also been described as a mild technique to solubilize IBs with efficient subsequent refolding. Also, in this case the presence of a denaturing agent at low concentrations (2 M urea) is required for efficient refolding [13–15]. The concomitant use of physical and chemical treatments, i.e., high hydrostatic pressures and alkaline pH, is described in the present study for the solubilization and subsequent refolding of protein aggregates.

ZIKV is a single-stranded RNA virus of the Flaviviridae family [16]. ZIKV genome is composed of 2 non-coding regions (5 ‘and 3’) flanking a region encoding a polyprotein that is cleaved into three structural proteins and 7 non-structural proteins (NS1, NS2A, NS2B, NS3, NS4A, NS4B and NS5) [17]. The NS1 proteins of flavivirus present a molecular mass of 46 to 55 kDa depending on the degree of glycosylation and six intramolecular disulfide bonds [18, 19]. The secondary structure consists of 21 β-sheets and two α-helices and the protein has three distinct domains: a N-terminal β-roll, an epitope-rich wing domain and a C-terminal β-ladder [20]. Flavivirus NS1 are proteins that associate with cell membrane lipids forming homodimers exposed in the membrane of the host cells. Recombinant NS1 produced by mammalian or insect cells are dimers that are converted to monomers by heating but not by SDS treatment [20–22]. Three dimeric NS subunits form soluble hexamers held together by weak hydrophobic interactions, a form that is readily dissociated into dimeric subunits in the presence of SDS [21, 23]. Flavivirus NS1, expressed by recombinant *E. coli,* appears however mainly as a monomer in SDS-PAGE [24–29], even without heating [24, 27].

NS1 is one of the most used markers for the detection of acute or convalescent infection by flaviviruses [22, 30]. This protein is used as an antigen in serological tests and has also interesting features for use in subunit vaccines [31]. Thus, production of a ZIKV NS1 with preserved structural and antigenic features may find different biotechnological applications.

In the present study, we applied HHP and alkaline pH for solubilization of NS1-IBs without chaotrope addition, allowing the efficient refolding of the ZIKV NS1 protein with preserved antigenic properties.

## Results

### Solubilization of NS1-IB

**Fig. 1:**
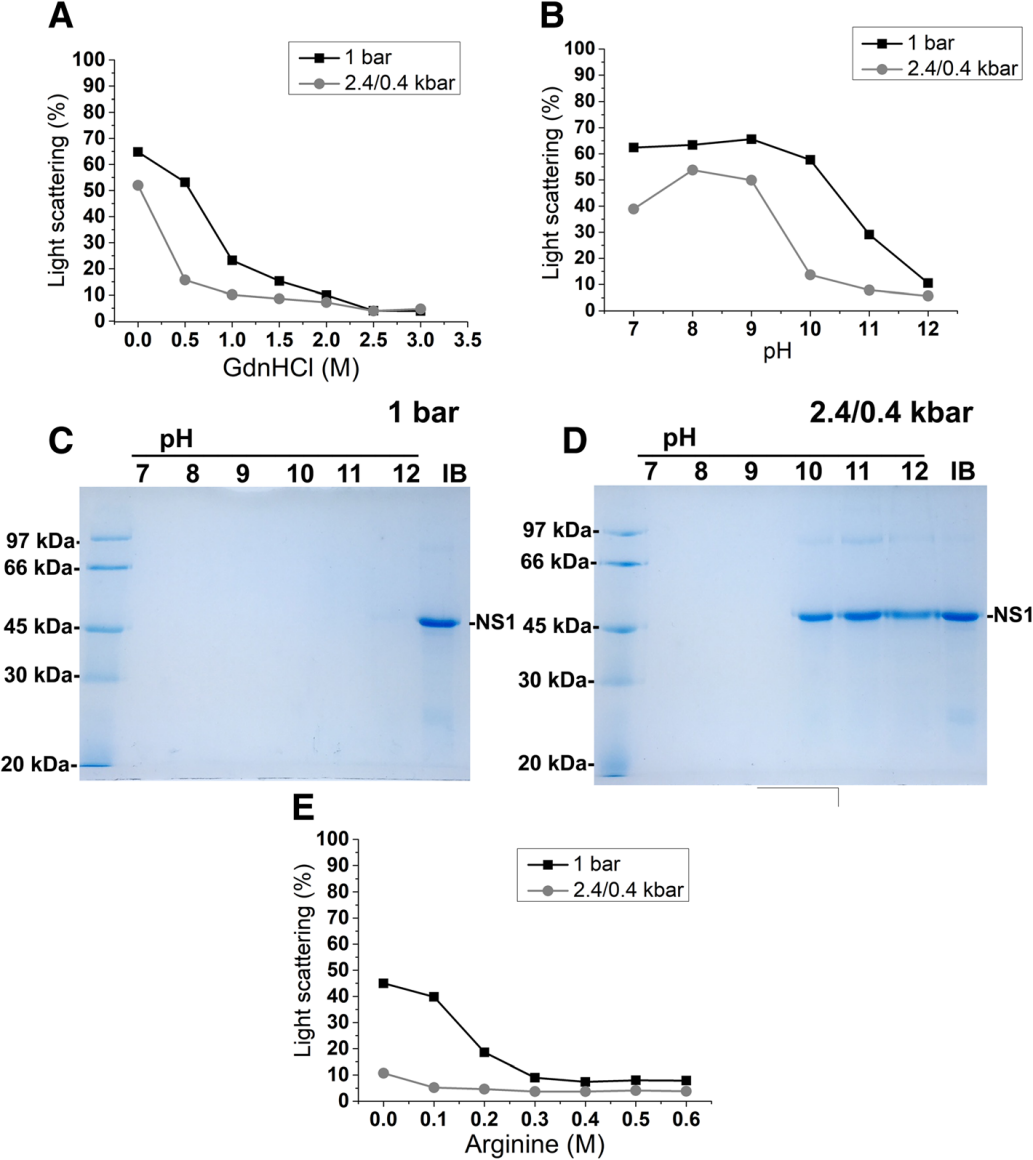
*Incubation at high pressure promotes solubilization of NS1-IBs.* Suspension of NS1-IBs was subjected to 2.4 kbar for 90 min and at 0.4 kbar for 14 h 30 min (2.4/0.4 kbar) or at 1 bar for 16 h. **a** Curve of LS vs GndHCl; **b** curve of LS vs pH; **c** SDS-PAGE analysis of the supernatant of the suspension subjected to 1 bar; **d** SDS-PAGE analysis of the supernatant of the suspension maintained at 2.4 kbar/0.4 kbar and **e** curve of LS vs Arg (pH 10.5). The LS measurements were carried out in a spectrofluorimeter with an excitation of 320 nm; the emission was determined between 315 and 325 nm, and the areas of the peaks were used for plotting. The LS value obtained for the suspension at a pH of 8.0 read immediately at 1 bar was considered to be 100%. For SDS-PAGE samples were boiled and reduced. The results are representative of experiments performed three times

The first step in establishing an efficient protein refolding protocol is the efficient solubilization of protein aggregates. To determine whether the association of HHP and guanidine hydrochloride (GndHCl) promote efficient solubilization of ZIKV NS1-IBs, suspensions were submitted to 2.4 kbar for 90 min and to 0.4 kbar for 14 h 30 min (2.4 kbar/0.4 kbar). We used this protocol since we have previously demonstrated that incubation at 2.4 kbar is sufficient for solubilization of aggregates, and incubation at lower pressure levels (0.35–0.7 kbar) is useful since refolding can occur and reaggregation is still impaired [10, 32]. As a control, the suspensions were maintained at atmospheric pressure (1 bar) for 16 h (Fig. 1a). The decrease in light scattering (LS) values at 320 nm, which indicates aggregate solubilization, was more evident for NS1-IB suspensions subjected to high pressure than those kept at 1 bar. The association of HHP with 1 M GdnHCl (at pH 8.5) enabled solubilization of ZIKV NS1-IB, as shown by the decay of more than 90% in LS values. To determine if association of alkaline pH and HHP was also capable to solubilize the NS1-IBs, suspensions were subjected to HHP or at 1 bar at a pH within the range of 7.0 to 12.0. As shown in Fig. 1b, LS decay was evident in suspensions incubated at HHP and at a pH of 10.0 and higher and the decay in LS values was lower for suspensions incubated at 1 bar. Recombinant ZIKV NS1 presents a molecular mass of 44,735 Da. The presence of NS1 band cannot be detected in the supernatants of the suspensions in the SDS-PAGE (Fig. 1c), indicating that dissociation of NS1-IBs at atmospheric pressure was not efficient. The presence of NS1 was observed, however, in the supernatant of the samples subjected to HHP at pH values 10 to 12 (Fig. 1d).

Considering the use of Arg in the refolding process of other proteins [18, 24, 33], we also tested this amino acid for the solubilization of ZIKV NS1. Protein solubilization at pH 10.5 was efficient even in the absence of Arg (Fig. 1e), as expected. However, the presence of this amino acid, even at a lowest concentration (0.1 M), further aided the solubilization of NS1-IBs subjected to high pressure (Fig. 1e).

### Analysis of solubilized NS1

**Fig. 2:**
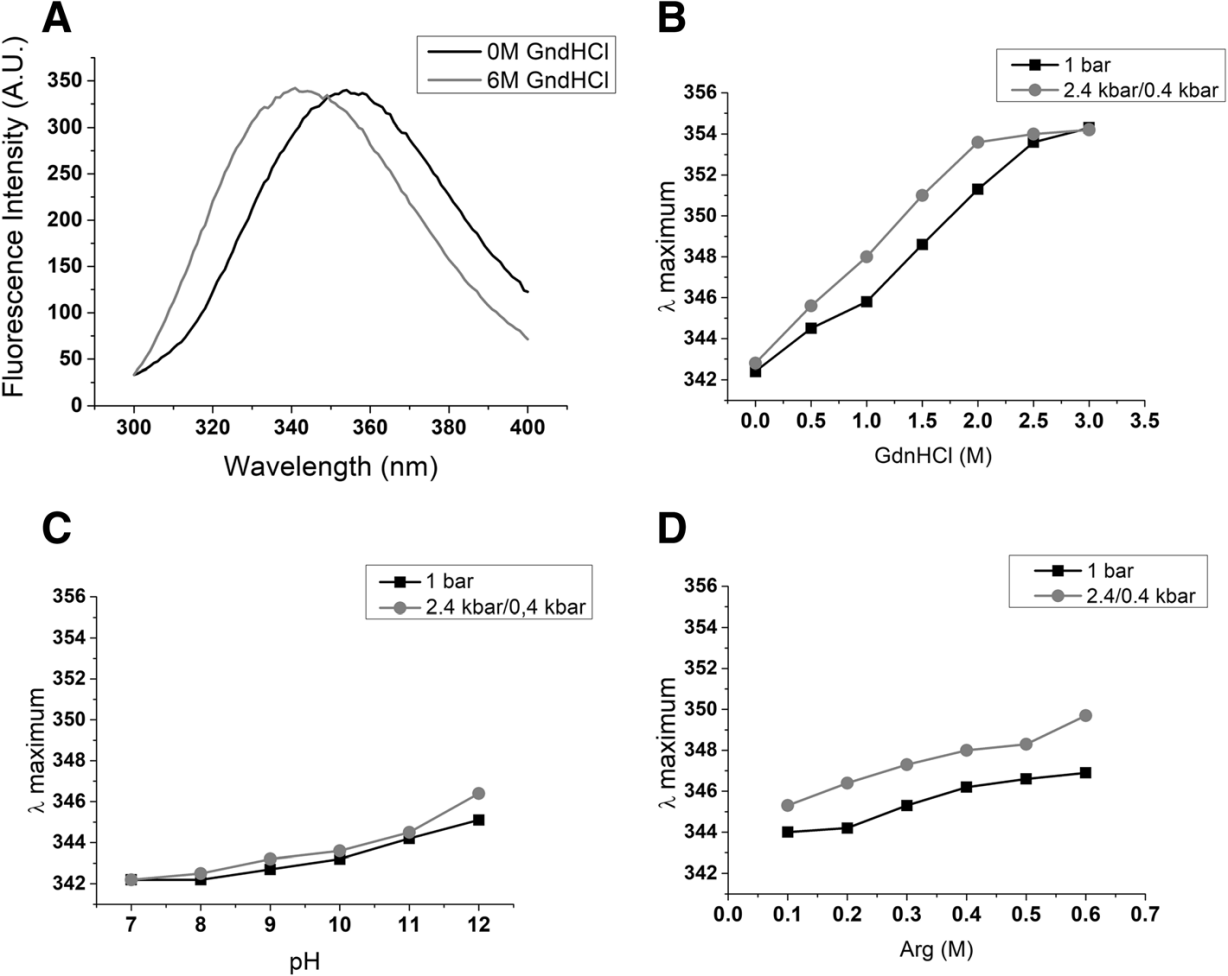
*High pressure in association with alkaline pH and the presence of Arg induce only partial unfolding of NS1-IB.* Suspensions of NS1-IB were subjected at 2.4 kbar/0.4 kbar or at 1 bar. **a** Intrinsic (Trp) fluorescence of NS1-IB; **b** λ maximum of fluorescence vs concentration of GdnHCl; **c** λ maximum of fluorescence vs pH and **d** λ maximum of fluorescence vs concentration of Arg. An excitation at 290 nm was used for intrinsic fluorescence determination and the emission was measured between 300 and 400 nm. The results are representative of experiments performed three times

Tryptophan (Trp) is an intrinsic fluorescence sensor of protein conformational changes [34]. The ZIKV NS1 contains 14 Trp residues [35]. An intrinsic fluorescence peak with maximum intensity (λ maximum) at a wavelength of approximately 344 nm was described for DENV NS1 in its native conformation, while a wavelength shift to 355 nm was observed for chemical-induced denatured DENV NS1 [24]. To determine the degree of unfolding of ZIKV NS1 generated by treatments to solubilize the IBs, the shifts in the intrinsic fluorescence peaks were monitored. A λ maximum of 342.2 nm was observed for the suspension of NS1-IBs at a pH of 7.0 at 1 bar and a shift of 12.8 nm (to 354.0 nm) was observed when the IBs were denatured by the presence of 6 M GdnHCl (Fig. 2a). The shifts in λ maximum observed for the NS1-IB subjected to HHP in the presence of GdnHCl were more pronounced than the shifts obtained for the samples subjected to compression at alkaline pH (Fig. 2b and c). It is noteworthy that NS1 that was subjected to HHP in the presence of 2 M GdnHCl present a displacement of the λ maximum of 11.4 nm (to 353.6 nm), indicating a high degree of unfolding, while the shift of the samples subjected to HHP at a pH of 11.0 was only 2.3 nm (to 344.5 nm) relative to the untreated suspension (Fig. 2c). A displacement of 5.8 nm (to 348 nm) was obtained for the λ maximum of NS1-IBs subjected to pressure at a pH of 10.5 in the presence of 0.4 M Arg (Fig. 2d). These results indicate that NS1 solubilization at alkaline pH is accompanied only by partial unfolding.

**Fig. 3:**
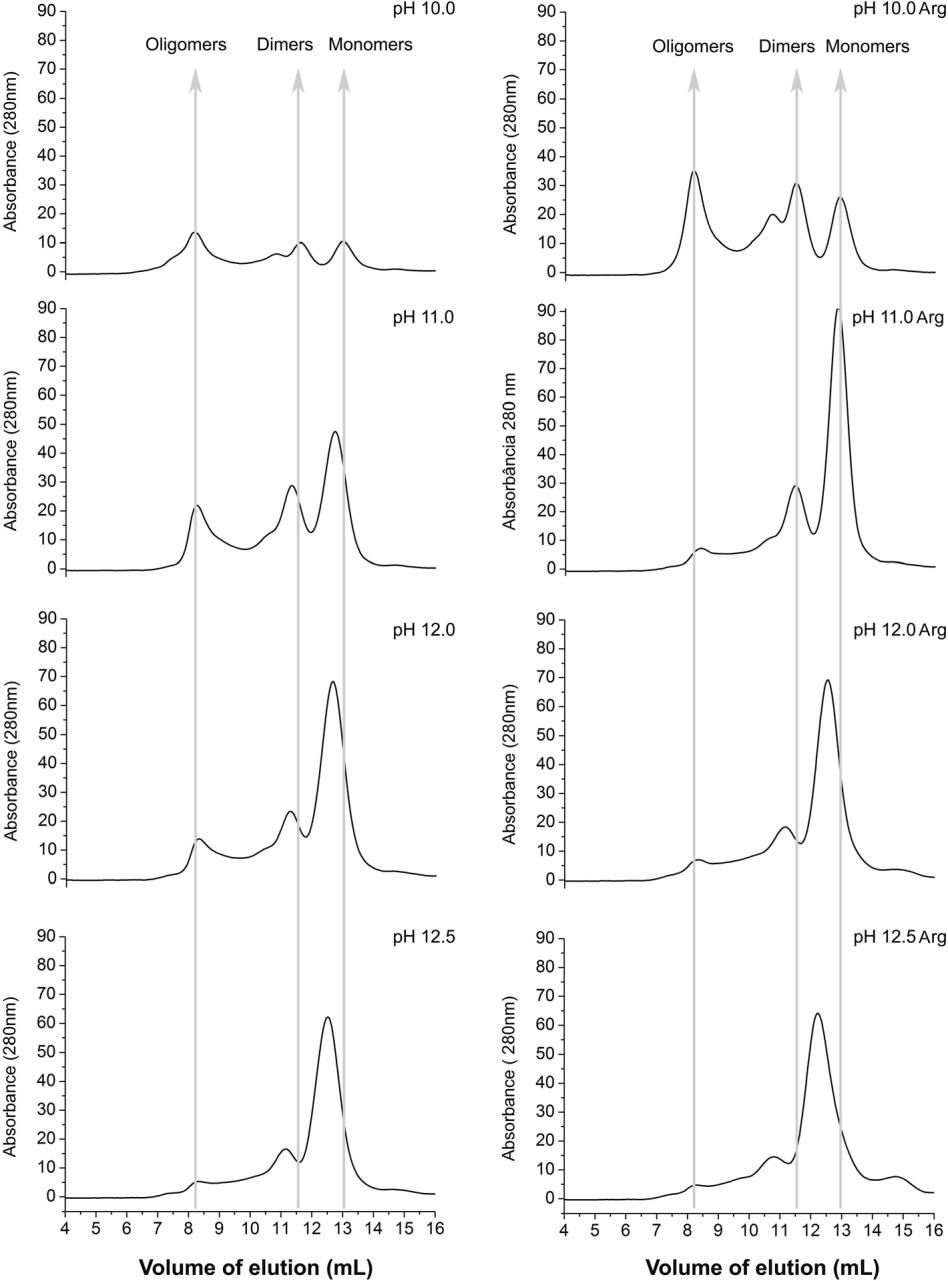
*Application of HHP at alkaline pH solubilizes NS1-IBs and the presence of Arg helps to dissociate oligomers.* SEC from supernatants of ZIKV NS1-IB suspensions subjected to 2.4 kbar/0.4 kbar. A volume of 500 μL of the supernatants of the suspensions subjected to HHP was applied to a Superdex 200 10/300 column (GE Biosciences). The elution buffer was 50 mM CAPS at a pH of 11.0

The analysis of the NS1 protein solubilized at alkaline pH and HHP was also performed by size exclusion chromatography (SEC). The chromatograms of Fig. 3 show the presence of peaks with retention volumes of approximately 8.4 mL, the column exclusion volume, which are probably soluble oligomers of NS1. The peaks presenting retention volumes of approximately 11.5 mL (86.5 kDa according to the linear regression equation obtained for SEC column calibration) are likely dimers, and the peaks that exhibit elution volumes of approximately 12.8 mL (52.6 kDa according to the linear regression equation) probably corresponding to ZIKV NS1 monomers. The peaks of NS1 solubilized at pH 10.0 in the absence of Arg have lower absorbance intensity than the samples treated with higher pH, suggesting that NS1 solubilization was not complete. Application of HHP to the IBs at pH from 11.0 to 12.5 solubilized the aggregates mainly into dimers and monomers. The presence of Arg contributed to the dissociation of NS1 oligomers. At higher pH, the volume of elution of NS1 monomers was slightly lower. The displacement was most evident at pH 12.0 and 12.5. Our explanation for this phenomenon is that the unfolding of the NS1 protein is higher at a more alkaline pH with increased volume of the protein. Thus, further solubilization attempts were carried out at pH lower than 12.0.

### Refolding of NS1

**Fig. 4:**
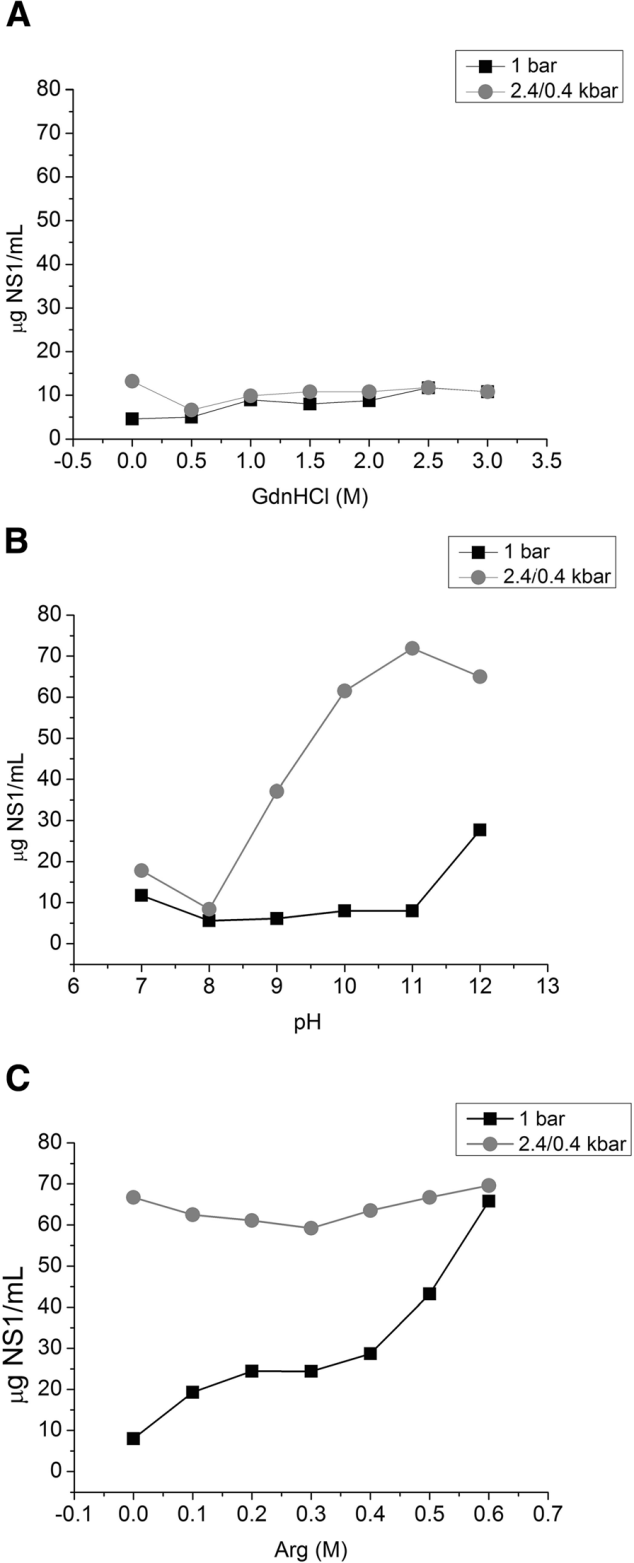
*Higher concentrations of NS1 are found in the supernatant of the suspensions subjected to HHP at alkaline pH.* Concentration of NS1 in ZIKV NS1-IB supernatants submitted to 2.4 kbar / 0.4 kbar or to 1 bar and dialyzed against 50 mM TrisHCl at a pH of 8.5. **a** concentration of NS1 vs concentration of GdnHCl; **b** concentration of NS1 vs pH; **c** concentration of NS1 vs concentration of Arg. The results are representative of experiments performed three times

NS1-IB suspensions subjected to HHP under the conditions shown in Figs. 1 and 2 were centrifuged, dialyzed to a lower pH (8.5), and the insoluble aggregates were removed by centrifugation. More than 6-fold higher levels of NS1 were observed for the samples subjected to HHP and alkaline pH than for the samples subjected to compression in the presence of GdnHCl, indicating lower degree of reaggregation after dialysis (Fig. 4). This result is possibly related to the lower degree of unfolding of these samples.

**Fig. 5:**
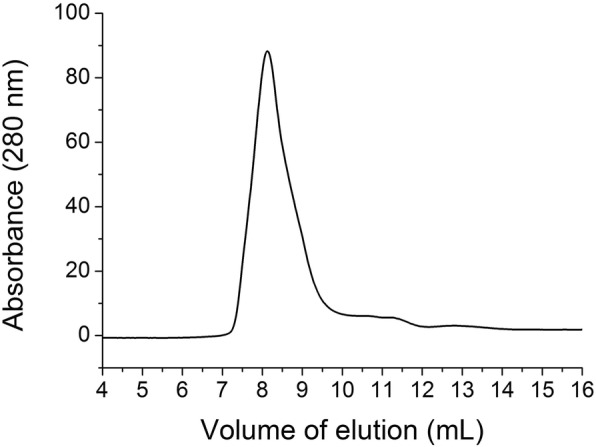
*NS1 solubilized by application of HHP at a pH of 11.5 forms oligomers after dialysis at a pH of 8.5.* ZIKV NS1-IB suspensions were subjected to 2.4 kbar/0.4 kbar at a pH of 11.5 and dialyzed against 50 mM Tris HCl at a pH of 8.5. A volume of 500 μL of the supernatant of the suspensions was applied to a Superdex 200 10/300 column (GE Biosciences). The elution buffer was TrisHCl 50 mM at a pH of 8.5

The supernatant of NS1-IBs subjected to HHP at pH 11.5 and dialysis to a pH of 8.5 was analysed by SEC. To our surprise, the protein eluted as a peak in a volume of 8.1 mL, which corresponds to the void volume of the column and a molecular mass of more than 500 kDa (Fig. 5). This result indicates that dimers/monomers of NS1 self-associated to form soluble oligomers upon dialysis to lower pH. ZIKV NS1 refolded at atmospheric pressure using an established protocol for DENV NS1 [27] was also observed mainly as oligomers in SEC (data not shown). Flavivirus NS1 are dimers that present a hydrophobic surface responsible for membrane association. The hydrophobic surface of ZIKV NS1 dimer is elongated in relation to the hydrophobic surface of West Nile virus NS1 [35]. Therefore, the property of ZIKV NS1 of forming oligomers can possibly be associated with its highly hybrophobic character.

**Fig. 6:**
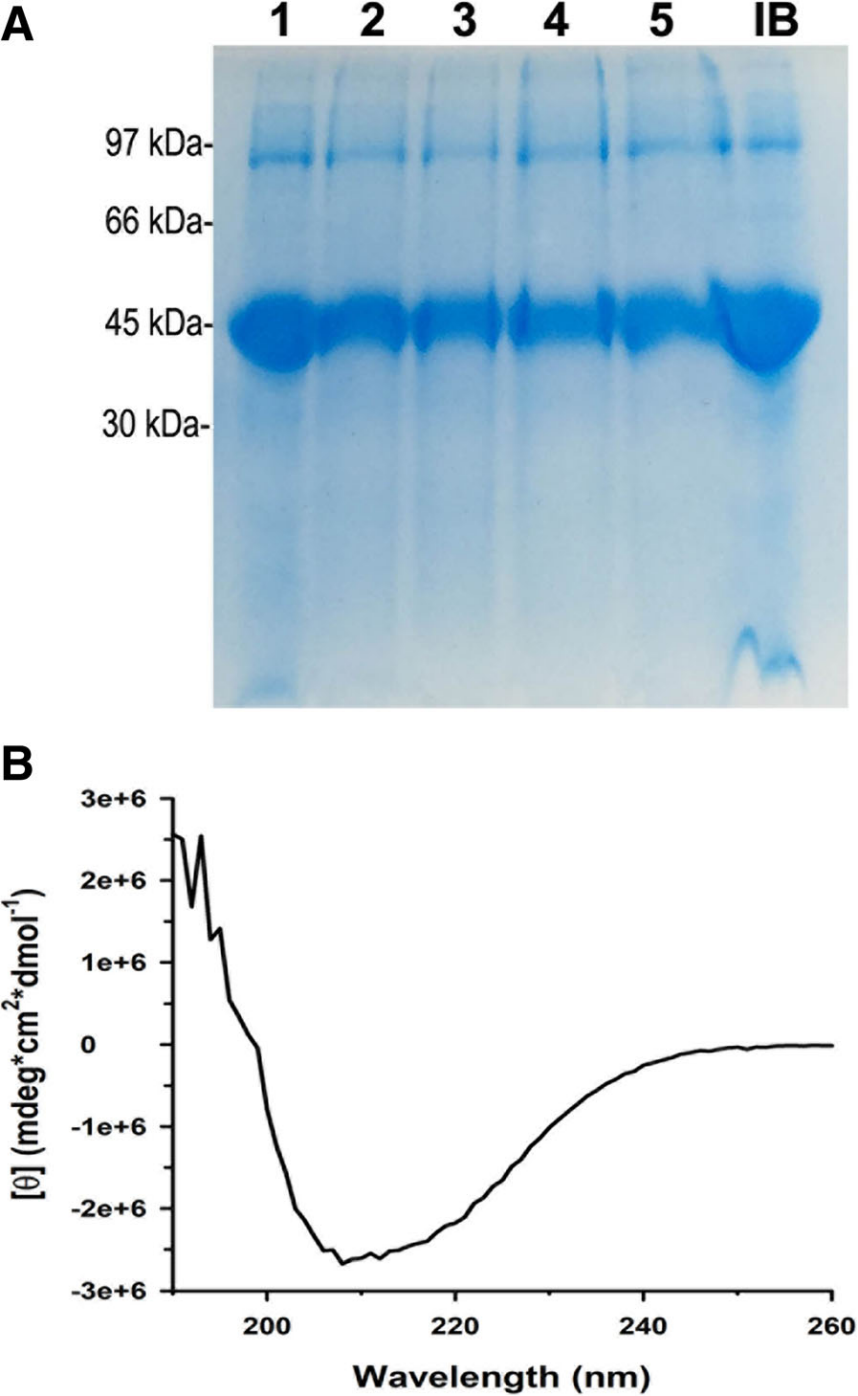
*Conformational analysis of refolded NS1.* **a** Supernatants from suspensions of NS1-IBs refolded by the application of HHP (2.4 kbar / 0.4 kbar) were dialyzed and analyzed by SDS-PAGE. Condition 1: pH 11.0 + DTT (2 mM); condition 2: pH 11.0 + Arg (0.4 M), condition 3: pH 11.0 + Arg + GSH (1 mM) + GSSG (0.1 mM); condition 4: pH 11.5 + Arg; condition 5: pH 11.5 + Arg + GSH/GSSG. Sample IB: The suspension was homogenized for application in the SDS-PAGE. All the samples were heated at 95 °C in the presence of DTT (100 mM) before application in SDS-PAGE; **b** Far-UV CD spectra of NS1 (2 μM recorded in 0.2 cm path length quartz cell) refolded at pH 11 in the presence of Arg

To determine the yield of refolded NS1 after dialysis to pH 8.5, supernatants of the suspensions subjected to HHP were compared to the same volume of the original NS1 IB suspension in SDS-PAGE. Bands with similar intensity were obtained for soluble NS1 and the protein in the NS1-IBs (Fig. 6a). The NS1 solubilized at HHP and a pH of 11.0 and 11.5 has a yield between 76 and 89% of the protein present in the NS1-IBs. The NS1 obtained was not purified, but it is 90–95% pure, as determined by the analysis of the image shown in the Fig. 6a. This high purity is due to the extensive washing of the NS1-IBs to eliminate soluble bacterial contaminants. The presence of the redox pair reduced (GSH) and oxidized (GSSG) glutathiones, which is frequently used to improve the formation of disulfide bonds in refolding protocols [36] or of the reducing reagent dithiothreitol (DTT) was also analyzed in relation to the refolding at pH 11 in the presence of Arg (Fig. 6a), with similar yields.

The recovery yield of refolded NS1 from IBs subjected to HHP either at pH 11.0 or 11.5 in the presence of 0.4 M arg reached up to 68 mg NS1/ per liter of bacterial culture. Such recovery yield is 27.2-fold higher than the yield (2.5 mg/L) obtained by refolding of NS1-IBs at atmospheric pressure using the protocol established for DENV NS1 refolding [27].

Circular dichroism spectrum (far-UV) of NS1 solubilized at pH 11.0 in the presence of Arg and refolded by dialysis indicates that the protein is recovered with its native conformation (Fig. 6b). Percentages of secondary structure components of NS1 protein were obtained by fitting the experimental CD spectrum. A total content of 14.6 ± 3.5% α-helices and 34.2 ± 2.3 β-sheets was obtained, which is in accordance with the 14% α-helices and 30% β-sheets present in ZIKV NS1 (PDB 5GS6).

### Reactivity of ZIKV-positive human serum against refolded NS1

**Fig. 7:**
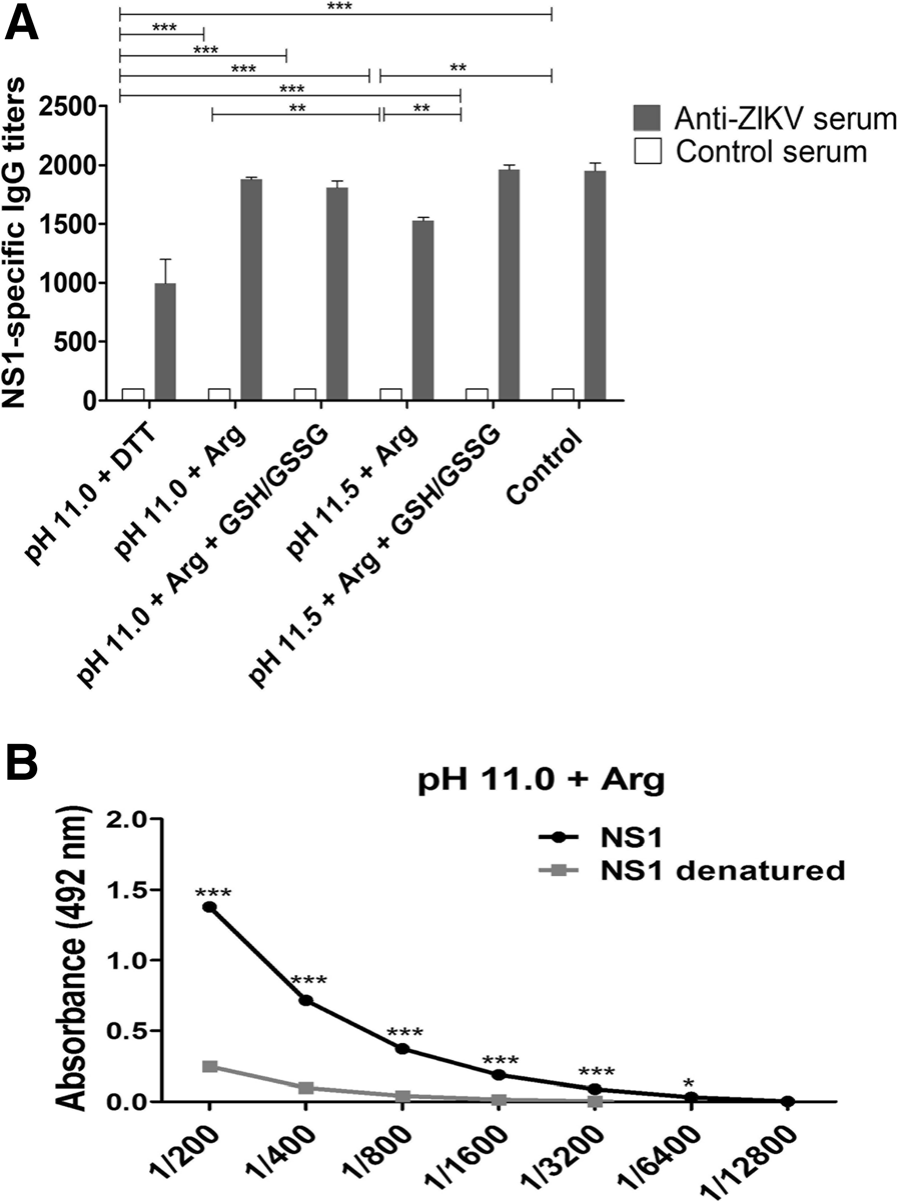
*NS1 of ZIKV refolded at HHP and pH of 11.0 is antigenic.* **a** NS1 was used as a solid phase antigen in ELISA assays employing control sera obtained from patients that had been previously infected with ZIKV (black bars) or not (white bars). Values are expressed as mean ± error of antigen-specific IgG antibody titers; **b** Evaluation of the preservation of conformational epitopes of the ZIKV NS1. NS1 obtained at HHP and pH 11.0 was submitted to denaturation (heating at 100 °C for 10 min followed by heat shock at 0 °C) or not and analyzed by ELISA for reactivity with a serum from a patient previously infected with ZIKV. The values obtained are expressed as mean ± error of the absorbance obtained in the assay. * *p* < 0.05; ** *p* < 0.01; *** *p* < 0.001 (Two-way ANOVA with Bonferroni test). The IB compression was performed in 2.4 kbar / 0.4 kbar. Control: NS1 obtained from the same clone used in this study and refolded using traditional protocol at atmospheric pressure

To determine the antigenicity of solubilized NS1, we performed ELISA tests with this antigen and serum samples from ZIKV-positive patients. The samples refolded at HHP presented high antibody titres with ZIKV-positive sera and low reactivity with sera from non-infected subjects. The titres were similar to those achieved with NS1 refolded with the established protocol carried out at atmospheric pressure (Fig. 7a). The presence of DTT (2 mM) during compression was shown to induce little enhancement of NS1 refolding yield (Fig. 6), but led to a decay of the NS1 antigenicity and the presence of glutathione pair did not significantly improve the antigenicity of the NS1. Higher titres of ZIKV-positive antibodies were obtained for refolded NS1 (black lines in Fig. 7b) than those obtained with the same protein that had been denatured by heating (gray lines in Fig. 7b), which suggests that the refolded protein preserves conformational epitopes.

## Discussion

The present study describes a solubilization method, based on HHP and alkaline pH, in which, at rather mild solubilization conditions, the ZIKV NS1 was recovered from IBs expressed in bacterial cells at high recovery yields and preserved immunological features. The combination of alkaline pH, that prompt the disruption of polar interactions induced by electrostatic repulsion [37] and HHP, that induce rupture of intermolecular hydrophobic interactions [5], proved to be quite efficient and led to the solubilization of insoluble aggregates with a low degree of protein unfolding. The process resulted in 6-fold higher yields of soluble protein than the achieved by association of HHP with GdnHCl. The final yield of refolded ZIKV NS1 was approximately 30-fold higher than a previously reported standard refolding method for DENV NS1 [27]. The refolding process of ZIKV NS1 using HHP and alkaline pH involves only two steps that, altogether, take less than 24 h. The refolding process was accomplished with the protein at a relatively high concentration (above 0.4 mg/mL) with final yields of approximately 70 mg/liter of bacterial culture. There was no need for dilution of the protein to avoid reaggregation and, consequently, no need for further protein concentration steps, usually required in protein refolding processes at atmospheric pressure. The refolded protein did not require further purification steps since it was recovered in a rather good purity (from 90 to 95%), which further contributes for reduced final costs and labor effort of the process.

ZIKV NS1 refolding has not been previously described. The refolding of the DENV NS1 required the use of 8 M urea for IB solubilization [24, 25, 27, 29]. Notably, the reported recovery yields were lower than the values achieved in the present study with ZIKV NS1-IBs (from 75 to 90%). The higher yields previously reported by Das et al. [25] were 60% and by Allonso et al. [24] that obtained a yield of 67% immunologically active NS1 from DENV NS1-IBs. In addition, all these processes are more time-consuming and laborious than the one described in this study.

The reactivity of human anti-ZIKV antibodies with purified ZIKV NS1 was comparable with the one obtained with the protein obtained using a protocol of IB solubilization with 8 M urea followed by metal affinity chromatography purification and refolding by further dilution [27]. Sera from ZIKV-infected subjects showed similar reactions with proteins obtained by the two methods. These results indicate that proteins obtained by the use of HHP in association with alkaline pH can be successfully applied in the generation of recombinant proteins for immunodiagnostic assays and vaccine development.

## Methods

### Expression of recombinant proteins, bacterial lysis and NS1-IB washes

For the expression of the ZIKV NS1 protein, the plasmid pETNS1ZIKV containing the sequence of the ZIKV NS1 (residues 797–1148) protein (access genbank: ALU33341.1) in the vector pET28a was designed and obtained commercially from GenScript (USA). This plasmid contains the gene that codifies for the full-length protein with 395 residues and two histidine tags at the N- and C-terminal residues. *Escherichia coli*, strain BL21 (DE3) (Novagen EMD Biosciences, Inc.), was used as host. The bacterial cultures for protein expression were grown in LB medium and expression induction was performed by addition of 0.5 mM IPTG when cultures reached an optical density (at 600 nm) of 0.5, as described [27]. Activated cultures (1 l) were centrifuged at 8000 x g for 10 min at 4 °C. The precipitated pellet was resuspended in 50 mL of buffer A1 (0.1 M Tris-HCl pH 8.5 + 5 mM EDTA). Lysozyme (50 μg / mL) was then added to the suspension and incubated at room temperature for 15 min. Sodium deoxycholate was then added to reach a concentration of 0.1%. Bacterial lysis was performed by sonicating the suspension on ice to avoid heating until the solution lost viscosity and was then centrifuged at 4000 x g for 10 min at 4 °C. The supernatant was discarded. The pellet was resuspended in 50 mL of buffer A2 (0.1 M Tris-HCl pH 8.5 containing 5 mM EDTA and 0.1% sodium deoxycholate), and the suspension was sonicated rapidly to disrupt the lumps. The suspension was centrifuged at 8000 x g for 10 min at 4 °C, and NS1-IBs was washed again. The suspension was washed once with 0.1 M Tris HCl buffer at pH 8.5 containing 1 mM EDTA and resuspended in 10–20 mL of the same buffer. The absorbance was read on a spectrophotometer at 350 nm, and the suspensions were separated into 1 mL aliquots that were kept in a freezer (− 20 °C) until time of use.

### High hydrostatic pressure

The optical density of the suspension of NS1-IBs was determined by spectrophotometer at 350 nm and diluted in the appropriate buffer containing 1 mM EDTA to optical density of 1.0, 2.0 or 5.0, which corresponds to approximately 0.08, 0.16 and 0.4 mg NS1/mL, respectively. The buffers used were 50 mM Tris HCl for pH 7.0 to 9.0 and 50 mM CAPS for pH 10.0 to 12.0. Arg generates an alkaline character (pH 11.1) in aqueous solution. Therefore, buffers were prepared by adding Arg to Tris HCl and titulated to the adequate pH. The suspensions of IBs were maintained at 1 bar for 16 h or plastic bags were filled with the samples and further sealed and included inside a larger plastic bag that was vacuum sealed and placed in the pressure vessel (R4–6-40, High Pressure Equipment). The vessel was pressurized at 2.4 kbar using suitable high-pressure pump (PS-50, High Pressure Equipment) using oil as a transmission fluid and was incubated in this condition for 90 min. The decompression was performed slowly until 0.4 kbar or to 1 bar. After decompression, the samples were centrifuged at 11,000 x g for 15 min to remove insoluble aggregates and dialyzed against 50 mM Tris HCl buffer at a pH of 8.5 to lower the pH and for removal of additives. The solution was centrifuged again and stored at − 20 °C for further analysis.

### Fluorescence and light scattering (LS)

Fluorescence and LS measurements were performed on a Cary Eclipse (Varian) spectrofluorimeter. Data were collected using 1 cm optical path cuvettes, and the measures were performed at a 90° angle relative to the incident light using a 1 s response time and reading speed of 240 nm/minute. LS measurements were performed with excitation at 320 nm, and scattering was collected from 315 to 325 nm. The intrinsic fluorescence emission of tryptophan (Trp) was collected between 300 and 400 nm, with excitation at 290 nm. LS curves were produced by increasing concentrations of GndHCl (0 to 3 M) or Arg (0 to 0.6 M) or increasing the pH (7 to 12).

### Polyacrylamide gel electrophoresis (SDS-PAGE)

SDS-PAGE was performed using 12% gel containing SDS and stained with Coomassie Blue G-250 [38]. The IB suspensions were heated at 95 °C for 5 min in sample buffer (50 mM Tris-HCl pH 8.5 and 0.01% bromophenol). For protein analysis in the reducing condition, the sample buffer also contained 100 mM dithiothreitol (DTT). Determination of the intensity of the bands of NS1 was performed by the analysis of the image using the ImageJ program.

### Size exclusion chromatography (SEC) analysis

Analysis of NS1 samples of ZIKV was performed on a Superdex 200 10/300 (GE Healthcare) SEC coupled to an AKTA (GE Healthcare) system. The buffer used for elution was Tris 50 mM at a pH of 8.5 or CAPS 50 mM at a pH of 11.0. Ovalbumin monomer (44.3 kDa), dimer (88.6 kDa) and bovine serum albumin monomer (66.4 kDa) were used to calibrate the column Superdex 200 10/300 (Additional file 1: Figure S1). We obtained a linear regression line of Y = − 0.03838 X + 14.82 (*N* = 3, r^2^ = 0.9989), where Y is the elution volume and X is the molecular mass.

### Circular dichroism (CD)

Circular dichroism measurements were carried out with a Jasco J-720 spectropolarimeter. Spectra were recorded in 0.2 cm path length quartz cells at a protein concentration of 2 μM in 10 mM phosphate buffer at pH 8.5. Three consecutive scans from 260 to 190 nm were performed. The observed raw ellipticities were converted into the molar ellipticities (θ). The secondary structure was estimated from fitted far-UV spectra using the DichroWeb server [39]. The total percentage of secondary structure components was obtained using different analysis programs (SELCON3, Contin-LL and CDSSTR) with different protein reference sets [40]. The results shown are the mean ± SD.

### Enzyme-linked immunosorbent assay (ELISA)

ELISAs were performed in 96-well plates. For plaque sensitization, 100 μL of NS1 diluted to 2 ng/μL in phosphate saline buffer (PBS) was applied to each well in duplicate. Plates were incubated at 4 °C for 16 to 18 h and washed in wash buffer (PBS containing 0.05% Tween-20). A volume of 200 μL of blocking buffer (PBS containing 0.05% Tween 20, 3% milk and 1% BSA) was added and incubated at 37 °C for 2 h. The blocking buffer was then discarded. Serum from patients positive or negative for ZIKV antibodies were diluted (PBS containing 0.05% Tween 20, 3% milk and 0.25% BSA) from 1: 200 to 1: 12,800, and 100 μL were added to each well and incubated at room temperature for 1 h. The wells were washed 3 times with 300 μL of wash buffer and 100 μL of peroxidase-conjugated goat anti-human IgG antibody (A0170, Sigma Aldrich) diluted 3000-fold in dilution buffer were added to each well. The plates were incubated at room temperature for 1 h. The plates were then washed 3 more times in wash buffer. The reaction was performed by the addition of the substrate, 0.04% of 1,2-diaminobenzene 1,2-phenylenediamine (OPD) and 0.04% hydrogen peroxide in 100 μL of 33 mM citrate-phosphate buffer at a pH of 5.5 to each well and incubated in the dark for 15 min at room temperature. The reaction was blocked by the addition of 50 μL of 1 M sulfuric acid. The plates were read in a spectrophotometer at 492 nm.

## Additional files

Additional file 1: **Figure S1.** Data used for calibration of SEC column. Volumes of elution of ovalbumin and BSA utilized for calibration of the column Superdex 200 10/300. (JPG 539 kb)

Additional file 2: Raw data of Figs. 1,2, 4 and 7. Data used for preparation of the Figs. 1, 2, 4 and 7. (DOCX 17 kb)

## Abbreviations

CD: Circular dichroism
DENV: Dengue virus
DTT: Dithiothreitol
EDTA: Ethylenediaminetetraacetic acid
ELISA: Enzyme-linked immunosorbent assay
GdnHCl: Guanidine hydrochloride
GSH: Reduced glutathione
GSSG: Oxidated glutathione
HHP: High hydrostatic pressure
IPTG: Isopropyl β-D-thiogalactopyranoside
LB: Luria-Bertani broth
LS: Light scattering
NS1: Non-structural protein 1
NS1-IB: Inclusion bodies of NS1
SDS-PAGE: Sodium dodecyl sulphate polyacrylamide gel electrophoresis
ZIKV: Zika virus

## References

[1] Ami D, Natalello A, Gatti-Lafranconi P, Lotti M, Doglia SM (2005). Kinetics of inclusion body formation studied in intact cells by FT-IR spectroscopy. FEBS Lett.

[2] Ami D, Natalello A, Taylor G, Tonon G, Maria Doglia S (2006). Structural analysis of protein inclusion bodies by Fourier transform infrared microspectroscopy. Biochim Biophys Acta.

[3] Peternel S, Grdadolnik J, Gaberc-Porekar V, Komel R (2008). Engineering inclusion bodies for non denaturing extraction of functional proteins. Microb Cell Factories.

[4] Rathore AS, Bade P, Joshi V, Pathak M, Pattanayek SK (2013). Refolding of biotech therapeutic proteins expressed in bacteria: review. J Chem Technol Biotechnol.

[5] Crisman RL, Randolph TW (2009). Refolding of proteins from inclusion bodies is favored by a diminished hydrophobic effect at elevated pressures. Biotechnol Bioeng.

[6] Silva JL, Oliveira AC, Vieira TCRG, de Oliveira GAP, Suarez MC, Foguel D: High-pressure chemical biology and biotechnology. Chem Rev 2014; 11414:7239–7267. 10.1021/cr400204z 24884274

[7] St John RJ, Carpenter JF, Balny C, Randolph TW (2001). High pressure refolding of recombinant human growth hormone from insoluble aggregates. Structural transformations, kinetic barriers, and energetics. J Biol Chem.

[8] St John RJ, Carpenter JF, Randolph TW (1999). High pressure fosters protein refolding from aggregates at high concentrations. Proc Natl Acad Sci U S A.

[9] St John RJ, Carpenter JF, Randolph TW (2002). High-pressure refolding of disulfide-cross-linked lysozyme aggregates: thermodynamics and optimization. Biotechnol Prog.

[10] Chura-Chambi RM, Cordeiro Y, Malavasi NV, Lemke LS, Rodrigues D, Morganti L (2013). An analysis of the factors that affect the dissociation of inclusion bodies and the refolding of endostatin under high pressure. Process Biochem.

[11] Chura-Chambi RM, Genova LA, Affonso R, Morganti L (2008). Refolding of endostatin from inclusion bodies using high hydrostatic pressure. Anal Biochem.

[12] Sen P, Ahmad B, Khan RH (2008). Formation of a molten globule like state in bovine serum albumin at alkaline pH. Eur Biophys J Biophy.

[13] Singh SM, Upadhyay AK, Panda AK (2008). Solubilization at high pH results in improved recovery of proteins from inclusion bodies of *Ecoli*. J Chem Technol Biotechnol.

[14] Patra AK, Mukhopadhyay R, Mukhija R, Krishnan A, Garg LC, Panda AK (2000). Optimization of inclusion body solubilization and renaturation of recombinant human growth hormone from *Escherichia coli*. Protein Expr Purif.

[15] Singh SM, Panda AK (2005). Solubilization and refolding of bacterial inclusion body proteins. J Biosci Bioeng.

[16] Lanciotti RS, Kosoy OL, Laven JJ, Velez JO, Lambert AJ, Johnson AJ, Stanfield SM, Duffy MR (2008). Genetic and serologic properties of Zika virus associated with an epidemic, yap state, Micronesia. 2007 Emerg Infect Dis.

[17] Kuno G, Chang GJ (2007). Full-length sequencing and genomic characterization of Bagaza, Kedougou. and Zika viruses Arch Virol.

[18] Edeling MA, Diamond MS, Fremont DH (2014). Structural basis of Flavivirus NS1 assembly and antibody recognition. Proc Natl Acad Sci U S A.

[19] Wallis TP, Huang CY, Nimkar SB, Young PR, Gorman JJ (2004). Determination of the disulfide bond arrangement of dengue virus NS1 protein. J Biol Chem.

[20] Akey DL, Brown WC, Dutta S, Konwerski J, Jose J, Jurkiw TJ, DelProposto J, Ogata CM, Skiniotis G, Kuhn RJ (2014). Flavivirus NS1 structures reveal surfaces for associations with membranes and the immune system. Science.

[21] Flamand M, Megret F, Mathieu M, Lepault J, Rey FA, Deubel V (1999). Dengue virus type 1 nonstructural glycoprotein NS1 is secreted from mammalian cells as a soluble hexamer in a glycosylation-dependent fashion. J Virol.

[22] Muller DA, Young PR (2013). The flavivirus NS1 protein: molecular and structural biology, immunology, role in pathogenesis and application as a diagnostic biomarker. Antivir Res.

[23] Gutsche I, Coulibaly F, Voss JE, Salmon J, d'Alayer J, Ermonval M, Larquet E, Charneau P, Krey T, Megret F (2011). Secreted dengue virus nonstructural protein NS1 is an atypical barrel-shaped high-density lipoprotein. Proc Natl Acad Sci U S A.

[24] Allonso D, da Silva Rosa M, Coelho DR, da Costa SM, Nogueira RM, Bozza FA, Santos FB, de Barcelos Alves AM, Mohana-Borges R (2011). Polyclonal antibodies against properly folded dengue virus NS1 protein expressed in *E. coli* enable sensitive and early dengue diagnosis. J Virol Methods.

[25] Das D, Mongkolaungkoon S, Suresh MR (2009). Super induction of dengue virus NS1 protein in *Ecoli*. Protein Expr Purif.

[26] Huang JL, Huang JH, Shyu RH, Teng CW, Lin YL, Kuo MD, Yao CW, Shaio MF (2001). High-level expression of recombinant dengue viral NS-1 protein and its potential use as a diagnostic antigen. J Med Virol.

[27] Amorim JH, Porchia BFMM, Balan A, Cavalcante RCM, da Costa SM, Alves AMD, Ferreira LCD (2010). Refolded dengue virus type 2 NS1 protein expressed in *Escherichia coli* preserves structural and immunological properties of the native protein. J Virol Methods.

[28] Sankar SG, Dhanajeyan KJ, Paramasivan R, Thenmozhi V, Tyagi BK, Vennison SJ. High-level expression of functionally active Dengue-2 non-structural antigen 1 production in *Escherichia coli*. Biomed Res Int. 2013. 10.1155/2013/343195 PMC3780544 24089673

[29] Athmaram TN, Saraswat S, Misra P, Shrivastava S, Singh AK, Verma SK, Gopalan N, Behara PK, Rao PV (2013). Optimization of Dengue-3 recombinant NS1 protein expression in *E. coli* and in vitro refolding for diagnostic applications. Virus Genes.

[30] Young PR, Hilditch PA, Bletchly C, Halloran W (2000). An antigen capture enzyme-linked immunosorbent assay reveals high levels of the dengue virus protein NS1 in the sera of infected patients. J Clin Microbiol.

[31] Amorim JH, Alves RP, Boscardin SB, Ferreira LC (2014). The dengue virus non-structural 1 protein: risks and benefits. Virus Res.

[32] Malavasi NV, Foguel D, Bonafe CFS, Braga CACA, Chura-Chambi RM, Vieira JM, Morganti L (2011). Protein refolding at high pressure: optimization using eGFP as a model. Process Biochem.

[33] Song H, Qi J, Haywood J, Shi Y, Gao GF (2016). Zika virus NS1 structure reveals diversity of electrostatic surfaces among flaviviruses. Nat Struct Mol Biol.

[34] Lacowicz JR. Principles of fluorescence spectroscopy**.** 3rd edn: Springer; 2006.

[35] Brown WC, Akey DL, Konwerski JR, Tarrasch JT, Skiniotis G, Kuhn RJ, Smith JL (2016). Extended surface for membrane association in Zika virus NS1 structure. Nat Struct Mol Biol.

[36] Clark ED (2001). Protein refolding for industrial processes. Curr Opin Biotechnol.

[37] Yongsawatdigul J, Park JW (2004). Effects of alkali and acid solubilization on gelation characteristics of rockfish muscle proteins. J Food Sci.

[38] Laemmli UK (1970). Cleavage of structural proteins during the assembly of the head of bacteriophage T4. Nature.

[39] Whitmore L, Wallace BA (2004). DICHROWEB, an online server for protein secondary structure analyses from circular dichroism spectroscopic data. Nucleic Acids Res.

[40] Sreerama N, Venyaminov SY, Woody RW (2000). Estimation of protein secondary structure from circular dichroism spectra: inclusion of denatured proteins with native proteins in the analysis. Nucleic Acids Res.

