# Supplementary figures and images for: Association of high pressure and alkaline condition for solubilization of inclusion bodies and refolding of the NS1 protein from zika virus
Source: BMC Biotechnol. 2018 Dec 12;18:78. doi: 10.1186/s12896-018-0486-2 (PMC6291932; doi:10.1186/s12896-018-0486-2)

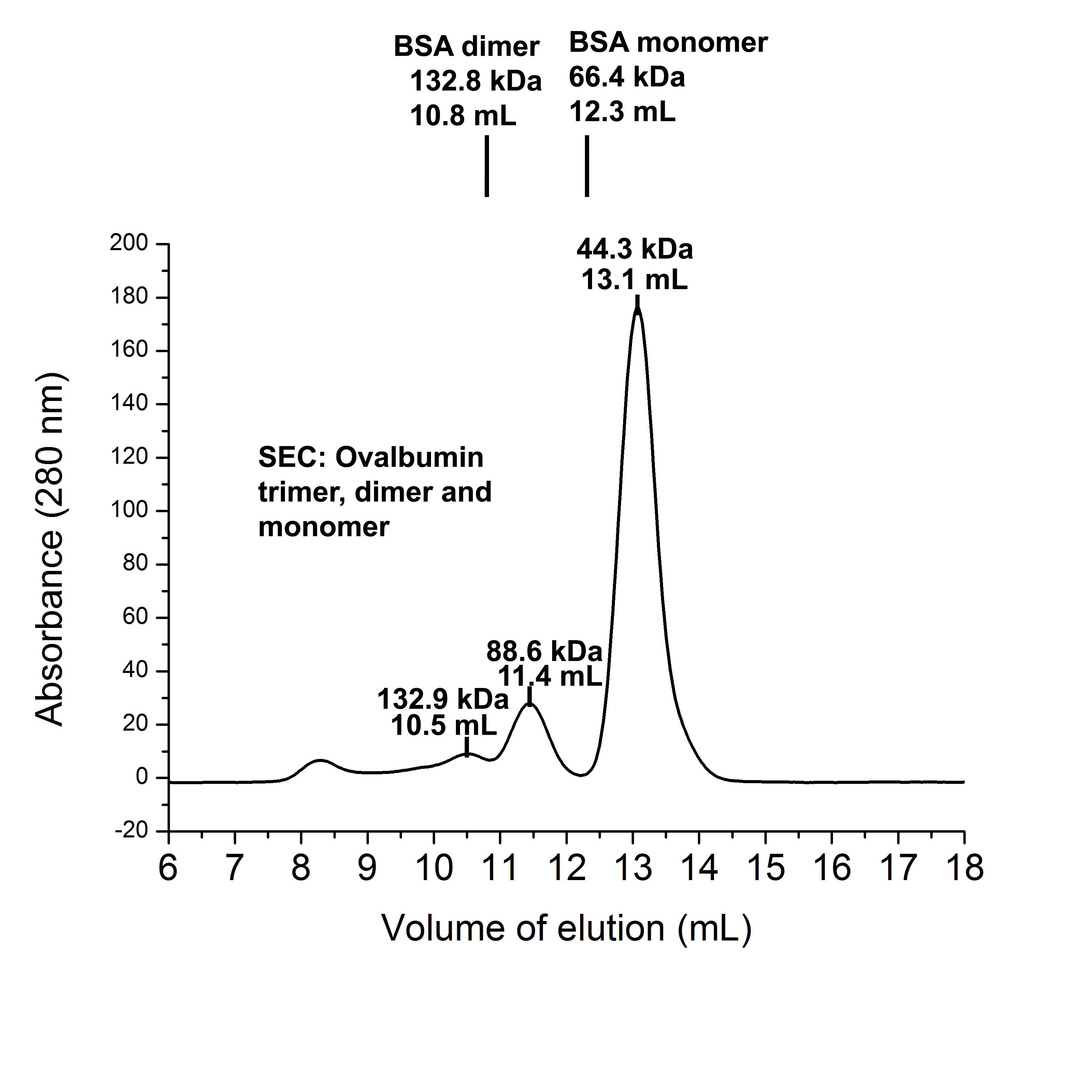

Supplement: Supplementary file 1 — Figure S1. Data used for calibration of SEC column. Volumes of elution of ovalbumin and BSA utilized for calibration of the column Superdex 200 10/300. (JPG 539 kb) [file 12896_2018_486_MOESM1_ESM.jpg]
