# Supplementary material for: Association of high pressure and alkaline condition for solubilization of inclusion bodies and refolding of the NS1 protein from zika virus
Source: BMC Biotechnol. 2018 Dec 12;18:78. doi: 10.1186/s12896-018-0486-2 (PMC6291932; doi:10.1186/s12896-018-0486-2)
Supplement: Supplementary file 2 — Raw data of Figs. 1,2, 4 and 7. Data used for preparation of the Figs. 1, 2, 4 and 7. (DOCX 17 kb) [file 12896_2018_486_MOESM2_ESM.docx]

**Additional Files**

**File 1. LIGHT SCATTERING (Fig 1A, B and E)**

**Figure 1A. LS x [GdnHCl]**

| **GdnHCl (M)** | **1 bar** | **2.4/0.4 kbar** |
| --- | --- | --- |
| 0 | 64.8 | 52.0 |
| 0.5 | 53.2 | 15.7 |
| 1.0 | 23.2 | 10.1 |
| 1.5 | 15.3 | 8.6 |
| 2.0 | 9.9 | 7.2 |
| 2.5 | 4.0 | 3.9 |
| 3.0 | 3.9 | 4.7 |

**Figure 1B. LS x pH**

| **pH** | **1 bar** | **2.4/0.4 kbar** |
| --- | --- | --- |
| 7 | 62.4 | 38.9 |
| 8 | 63.4 | 53.8 |
| 9 | 65.6 | 499 |
| 10 | 57.7 | 13.7 |
| 11 | 29.1 | 7.9 |
| 12 | 10.5 | 5.7 |

**Figure 1C. LS x [Arg]**

| **Arg (M)** | **1 bar** | **2.4/0.4 kbar** |
| --- | --- | --- |
| 0 | 45.0 | 10.7 |
| 0.5 | 39.8 | 5.2 |
| 1.0 | 18.6 | 4.6 |
| 1.5 | 9.0 | 3.7 |
| 2.0 | 7.4 | 3.7 |
| 2.5 | 8.0 | 4.1 |
| 3.0 | 7.9 | 3.8 |

**File 2. λ MAXIMAL (Fig 2B, C and D)**

**Figure 2B. λ max x [GdnHCl]**

| **GdnHCl (M)** | **1 bar**  **(nm)** | **2.4/0.4 kbar**  **(nm)** |
| --- | --- | --- |
| 0 | 342.4 | 342.8 |
| 0.5 | 344.5 | 345.6 |
| 1.0 | 345.8 | 348.0 |
| 1.5 | 348.6 | 351.0 |
| 2.0 | 351.3 | 353.6 |
| 2.5 | 353.6 | 354.0 |
| 3.0 | 354.3 | 354.2 |

**Figure 2C. λ max x pH**

| **pH** | **1 bar**  **(nm)** | **2.4/0.4 kbar**  **(nm)** |
| --- | --- | --- |
| 7 | 342.2 | 342.2 |
| 8 | 342.2 | 342.5 |
| 9 | 342.7 | 343.2 |
| 10 | 343.2 | 343.6 |
| 11 | 344.2 | 344.5 |
| 12 | 345.1 | 346.4 |

**Figure 2D. λ max x [Arg]**

| **Arg (M)** | **1 bar**  **(nm)** | **2.4/0.4 kbar**  **(nm)** |
| --- | --- | --- |
| 0 | 342.2 | 342.2 |
| 0.1 | 344.0 | 345.3 |
| 0.2 | 344.2 | 346.4 |
| 0.3 | 345.3 | 347.3 |
| 0.4 | 346.2 | 348.0 |
| 0.5 | 346.6 | 348.3 |
| 0.6 | 346.9 | 349.7 |

**File 3. NS1 CONCENTRATION: Fig 4A, B and C**

**Figure 4A. [GdnHCl] x concentration of NS1/mL**

| **GdnHCl (M)** | **1 bar**  **(µg NS1/mL)** | **2.4/0.4 kbar**  **(µg NS1/mL)** |
| --- | --- | --- |
| 0 | 4.55 | 0 |
| 0.5 | 4.97 | 0.5 |
| 1.0 | 8.92 | 1.0 |
| 1.5 | 7.98 | 1.5 |
| 2.0 | 8.73 | 2.0 |
| 2.5 | 11.7 | 2.5 |
| 3.0 | 10.8 | 3.0 |

**Figure 4B. pH x concentration of NS1/mL**

| **pH** | **1 bar**  **(µg NS1/mL)** | **2.4/0.4 kbar**  **(µg NS1/mL)** |
| --- | --- | --- |
| 7 | 11.7 | 17.8 |
| 8 | 5.6 | 8.4 |
| 9 | 6.11 | 37.1 |
| 10 | 7.99 | 61.5 |
| 11 | 7.99 | 71.9 |
| 12 | 27.7 | 65.0 |

**Figure 4C. [Arg] x concentration of NS1/mL**

| **Arg (M)** | **1 bar**  **(µg NS1/mL)** | **2.4/0.4 kbar**  **(µg NS1/mL)** |
| --- | --- | --- |
| 0 | 7.99 | 66.7 |
| 0.1 | 19.3 | 62.5 |
| 0.2 | 24.4 | 61.1 |
| 0.3 | 24.4 | 59.2 |
| 0.4 | 28.7 | 63.5 |
| 0.5 | 43.2 | 66.7 |
| 0.6 | 65.8 | 69.6 |

**File 4. ELISA Figure 7A and B**

**Figure 7A. ELISA. NS1-specific Ig-G titres**

|  | **Control sera**  **NS1-specific IgG titre** | | **Anti-ZIKV sera**  **NS1-specific IgG titre** | | **Media** ± SEM |
| --- | --- | --- | --- | --- | --- |
| pH 11.0 + DTT | 100 | 100 | 1198.2 | 790.4 | 994.3 ± 203.9 |
| pH 11.0 + Arg | 100 | 100 | 1860.7 | 1897.3 | 1879.0 ± 18.3 |
| pH 11.0 + Arg + GSH/GSSG | 100 | 100 | 1753.1 | 1866.6 | 1809.9 ± 56.7 |
| pH 11.5 + Arg | 100 | 100 | 1553.7 | 1500.6 | 1527.2 ± 26.6 |
| pH 11.5 + Arg + GSH/GSSG | 100 | 100 | 2002.1 | 1921.2 | 1961.6 ± 40.4 |
| Control | 100 | 100 | 1887.8 | 2017.3 | 1952.6 ± 64.7 |

**Figure 7B. Evaluation of preservation of conformational epitopes of ZIKV NS1.**

| **Dilution** | **NS1**  **Absorbance** | | **Media ± SEM** | **Denatured NS1**  **Absorbance** | | **Media ± SEM** |
| --- | --- | --- | --- | --- | --- | --- |
| 1/200 | 1.37225 | 1.38625 | 1.379 ± 0.007 | 0.2491667 | 0.2461667 | 0.2476 ± 0.001 |
| 1/400 | 0.74525 | 0.68625 | 0.716 ± 0.029 | 0.09516667 | 0.1011667 | 0.0981 ± 0.003 |
| 1/800 | 0.38425 | 0.36225 | 0.373 ± 0.011 | 0.05316667 | 0.02716667 | 0.0401 ± 0.013 |
| 1/1600 | 0.18825 | 0.18625 | 0.187 ±0.001 | 0.01816667 | 0.01016667 | 0.0141 ± 0.004 |
| 1/3200 | 0.08725 | 0.08925 | 0.088 ± 0.001 | 0.007166667 | -0.00083333 | 0.0032 ± 0.004 |
| 1/6400 | 0.03225 | 0.02925 | 0.0307 ± 0.001 | -0.01583333 | -0.01383333 | -0.0148 ± 0.001 |
| 1/12800 | 0.00125 | 0.00325 | 0.0225 ± 0.001 | -0.02383333 | -0.02083333 | -0.0223 ± 0.001 |
